# Rapid Detection of the Omicron (B.1.1.529) SARS-CoV-2 Variant Using a COVID-19 Diagnostic PCR Assay

**DOI:** 10.1128/spectrum.00990-22

**Published:** 2022-07-05

**Authors:** Chiara Ippoliti, Flavio De Maio, Giulia Santarelli, Simona Marchetti, Antonietta Vella, Rosaria Santangelo, Maurizio Sanguinetti, Brunella Posteraro

**Affiliations:** a Dipartimento di Scienze Biotecnologiche di Base, Cliniche Intensivologiche e Perioperatorie, Università Cattolica del Sacro Cuore, Rome, Italy; b Dipartimento di Scienze di Laboratorio e Infettivologiche, Fondazione Policlinico Universitario A. Gemelli IRCCS, Rome, Italy; c Dipartimento di Scienze Mediche e Chirurgiche, Fondazione Policlinico Universitario A. Gemelli IRCCS, Rome, Italy; Johns Hopkins Hospital

**Keywords:** PCR assay, Omicron SARS-CoV-2 variant, rapid testing

## Abstract

The Omicron (B.1.1.529) variant of severe acute respiratory syndrome coronavirus 2 (SARS-CoV-2) is the last variant of concern (VOC) identified to date. Compared to whole-genome or gene-specific sequencing methods, reverse-transcription PCR assays may be a simpler approach to study VOCs. We used a point-of-care COVID-19 diagnostic PCR assay to detect the Omicron SARS-CoV-2 variant in the respiratory tract samples of COVID-19 patients who had tested positive for SARS-CoV-2 RNA between April 2021 and January 2022. Sequencing analyses had shown that 87 samples were positive for the Omicron variant and 43 samples were positive for a non-Omicron variant (Delta, 18 samples; Alpha, 13 samples; Gamma, 10 samples; Beta, 1 sample; or Epsilon, 1 sample). According to results by the PCR assay, whose primers anneal a nucleocapsid (N) gene region that comprises the E31/R32/S33 deletion (also termed the del31/33 mutation), we found that N gene target failure/dropout (i.e., a negative/low result) occurred in 86 (98.8%) of 87 Omicron variant-positive samples tested. These results were assessed in relation to those of the spike (S) gene, which expectedly, was detected in all (100%) 130 samples. A total of 43 (100%) of 43 Delta, Alpha, Gamma, Beta, or Epsilon variant-positive samples had a positive result with the N gene. Importantly, in 86 of 87 Omicron variant-positive samples, the del31/33 mutation was detected together with a P13L mutation, which was, instead, detected alone in the Omicron variant-positive sample that had a positive N-gene result.

**IMPORTANCE** Rapid detection of the Omicron SARS-CoV-2 variant in patients’ respiratory tract samples may influence therapeutic choices, because this variant is known to escape from certain monoclonal antibodies. Our findings strengthen the importance of manufacturers’ efforts to improve the existing COVID-19 diagnostic PCR assays and/or to develop novel variant-specific PCR assays. Furthermore, our findings show that only a small fraction of SARS-CoV-2-positive samples may require whole-genome sequencing analysis, which is still crucial to validate PCR assay results. We acknowledge that the emergence of novel variants containing mutations outside the PCR assay target region could, however, allow an assay to work as per specifications without being able to identify a SARS-CoV-2-positive sample as a variant. Future work and more experience in this topic will help to reduce the risk of misidentification of SARS-CoV-2 variants that is unavoidable when using the current PCR assays.

## OBSERVATION

Since November 2021, almost 2 years after the severe acute respiratory syndrome coronavirus 2 (SARS-CoV-2) was identified as the etiological agent of coronavirus disease 2019 (COVID-19), the Omicron (B.1.1.529) SARS-CoV-2 variant has become notorious as one of the variants of concern (VOCs) with an unprecedented infectivity and transmissibility (1). Omicron (B.1.1.529) was split into two sublineages, named BA.1 and BA.2, until very recently, when two sister variants of BA.1 (BA.4 and BA.5) were recognized (2). While whole-genome sequencing is the current gold standard for SARS-CoV-2 variant identification (3), reverse-transcription PCR assays represent a simpler approach to study specific VOCs (4). Knowing a patient is infected by an Omicron variant may affect therapeutic choices, as this variant seems to escape from certain monoclonal antibodies (5, 6).

Compared to the Delta (B.1.617.2) SARS-CoV-2 variant that appeared in early 2021 (4), Omicron had a greater number (26 to 32) of nonsynonymous substitution or deletion mutations in the spike (S) protein and its receptor-binding domain (7). Of these mutations, the H69/V70 deletion (also termed the del69/70 mutation) was found to cause an S-gene target failure or S-gene dropout in (some) diagnostic PCR assays that target the S gene (7, 8). Despite being suggested as a marker for the Omicron variant, especially in contexts where the prevalence of Alpha (B.1.1.7) or subsets of Gamma (P.1) and Delta SARS-CoV-2 variants may be negligible (7), S-driven diagnostic failure may be circumvented when using multiplex PCR assays (8). Additionally, the Omicron sublineage BA.2 (not carrying the del69/70 mutation) does not show S-gene dropout (7). Thus, as for Alpha or Delta variants (9–11), N-gene dropout, which may be qualified as a shift in the N-gene cycle threshold (*C_T_*) value compared to other target genes’ values, in multiplex PCR assays could be a valuable indicator of the Omicron variant. Consistently, Omicron variant-defining mutations, such as P13L, E31del, R32del, S33del, R203K, and G204R in the N protein, could lead to N-gene target failure in PCR assays that target the N gene.

We used a point-of-care COVID-19 PCR assay, namely, the AIGS COVID-19 nucleic acid (RNA) POCT detection kit (Life Real; Alifax, Padua, Italy) to screen respiratory tract samples from COVID-19 patients for the Omicron variant. The assay, marketed before November 2021 (12), uses PCR primers that anneal a nucleocapsid (N) gene region that comprises the E31/R32/S33 deletion (also termed the del31/33 mutation), which has shown 95.1% presence in the sequences of all Omicron variant (BA.1 through BA.5) sublineages (13). Due to intellectual property concerns, the manufacturer did not disclose detailed information on its assay’s diagnostic (S and N) targets, including the sequences of the primers for the SARS-CoV-2 RNA PCR-based amplification (https://www.medicalliance.global/vis-content/event-medcom2020.MEDICA/exh-medcom2020.2676659/MEDICA-2020-Hangzhou-Lifereal-Biotechnology-Co.-Ltd-Product-medcom2020.2676659-KrmM2hF8QhuRx7KWtEitCg.pdf). However, in the presence of the Omicron variant, an unaffected S-gene detection along with an N-gene target failure (or N-gene dropout) should be expected.

We included residual samples from nasopharyngeal swabs (*n *= 130) that had been obtained, each from a single COVID-19 patient, between April 2021 and January 2022. Samples had resulted positive (*C_T_* value, ≤25 of the envelope [E] gene) at SARS-CoV-2 diagnostic PCR testing (i.e., performed using an updated version of the Seegene Allplex 2019-nCoV assay [14], which was designated the Allplex SARS-CoV-2 assay). As an established preanalytical criterion by the Lazio Italian Region SARS-CoV-2 genome-surveillance project participants, including us (https://www.salute.gov.it/imgs/C_17_pagineAree_5453_27_file.pdf), a *C_T_* value of 25 represented the cutoff above which a percentage of genomic coverage of less than 90 (https://seqcovid.csic.es/evaluating-qpcr-cycle-threshold-ct-as-a-predictive-value-of-sequencing-quality) was likely to occur. While appearing very conservative, this cutoff is only slightly different from what has been suggested to optimize the sequencing success elsewhere (15). After immediate storage at −20°C, RNA extract-containing samples were subjected to S-gene sequencing (only for samples collected before 1 July 2021, using S-specific PCR primers and the Arrows Diagnostics SARS-CoV-2 S gene kit) or whole-genome sequencing (using the Illumina CovidSeq assay kit) analyses. Accordingly, 87 (66.9%) of 130 samples were positive for Omicron (B.1.1.529), 18 (13.8%) for Delta (B.1.617.2), 13 (10.0%) for Alpha (B.1.1.7), 10 (7.7%) for Gamma (P.1), 1 (0.8%) for Beta (B.1.351), and 1 (0.8%) for Epsilon (B.1.427), respectively. Of the 87 Omicron variants, 84 were BA.1 and 3 were BA.2. This sample collection was moderately representative of all the SARS-CoV-2 variants known and/or clinically encountered thus far (2).

When tested with the PCR assay (Table 1), all (100%) of the 87 Omicron variant-positive samples yielded a positive result with the S gene, whereas 86 (98.8%) of 87 samples yielded negative (*n *= 62) or low (*n *= 24) results with the N gene, results which were, respectively, consistent with an N-gene target failure or N-gene dropout. Specifically, in the 24 samples, PCR *C_T_* values for the N gene ranged from 24.19 to 35.31 (mean ± standard deviation [SD] *C_T_* value, 29.32 ± 1.91), whereas *C_T_* values for the S gene ranged from 9.04 to 23.53 (mean ± SD *C_T_* value, 13.64 ± 4.97). Therefore, for each of 24 samples, Δ*CT*_N−S_ (N-gene *C_T_* – S-gene *C_T_*) was ≥6.01. The remaining one sample with an N gene detected had a *C_T_* value of 21.21, which was almost identical to the S-gene *C_T_* value (20.84); thus, according to a Δ*CT*_N−S_ of <6.01, this sample was classified as having a positive N-gene result. Conversely, all (100%) of the 43 Delta-, Alpha-, Gamma-, Beta-, or Epsilon-positive samples yielded a positive result with both S and N genes. These results agreed with the presence of Omicron variant-defining mutations, including nonsynonymous substitutions (P13L, R203K, and G204R) or deletions (E31del, R32del, and S33del), which were, alone or in combination, identified in all (100%) of the 87 Omicron variant-positive samples (Table 1). All of the 87 samples had a P13L mutation, which was detected alone (one sample) or along with an E31del/R32del/S33del (86 samples) or R203K/G204R (75 samples) combination. The sample with only the P13L mutation detected gave a positive result with the N gene.

**TABLE 1:** PCR assay testing results for COVID-19 patients’ samples according to the presence of mutations in the SARS-CoV-2 nucleocapsid gene^*a*^

WHO label	Pango lineage	Total tested	S-gene detection (no.)	N-gene target-failure/dropout (no.)	No. of Omicron variant-defining mutations identified					
					E31del	R32del	S33del	P13L	R203K	G204R
Omicron	B.1.1.529	87	87	86	86	86	86	87	75	75
Delta	B.1.617.2	18	18	0	0	0	0	0	0	0
Alpha	B.1.1.7	13	13	0	NA	NA	NA	NA	NA	NA
Gamma	P.1	10	10	0	0	0	0	0	0	0
Beta	B.1.351	1	1	0	NA	NA	NA	NA	NA	NA
Epsilon	B.1.427	1	1	0	NA	NA	NA	NA	NA	NA

a Mutations in the SARS-CoV-2 nucleocapsid (N) gene were identified for samples (*n *= 115) collected after 1 July 2021, when whole-genome sequencing analysis has been implemented for SARS-CoV-2 variant surveillance purposes in the Lazio region of Italy, which is the study’s location. Before that date, only the SARS-CoV-2 spike (S) gene sequencing has been performed, and this regarded samples (*n *= 15) with a WHO-labeled SARS-CoV-2 Alpha, Beta, or Epsilon variant detected. Listed are nonsynonymous substitutions (P13L, R203K, and G204R) or deletions (E31del, R32del, and S33del), within the N-protein-encoding gene that define the WHO-labeled SARS-CoV-2 Omicron variant (2). COVID-19, coronavirus disease 2019; NA, not available; Pango, phylogenetic assignment of named global outbreak; SARS-CoV-2, severe acute respiratory syndrome coronavirus 2; WHO, World Health Organization.

**FIG 1:**
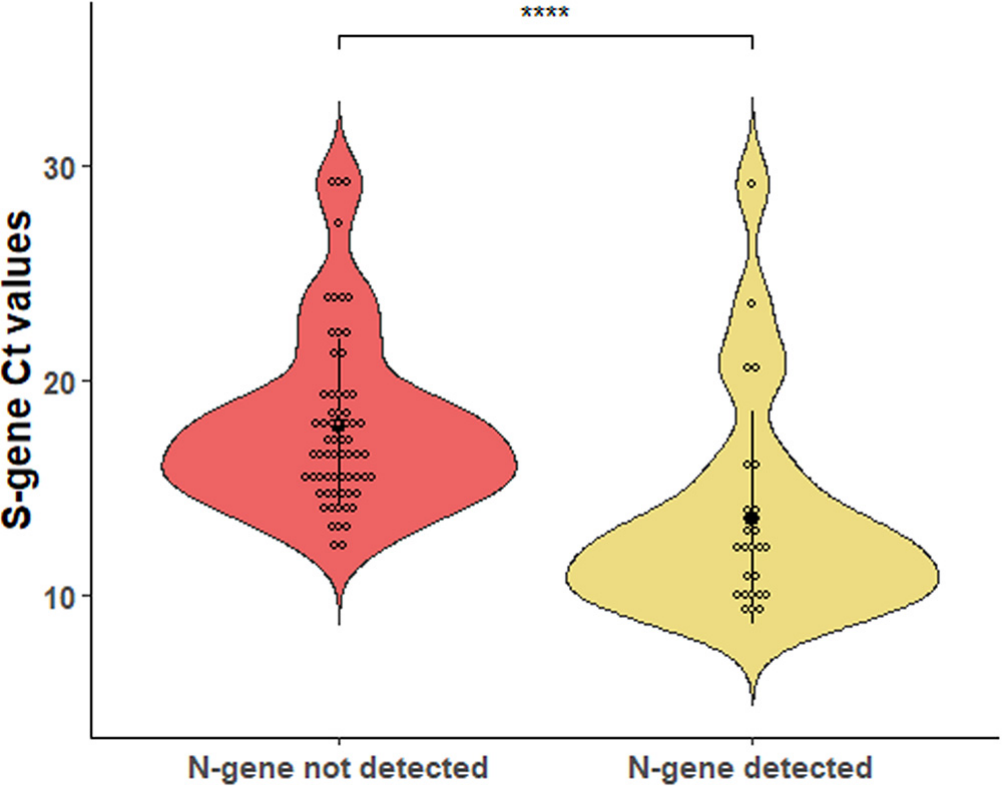
Distribution of cycle threshold (*C_T_*) values for the SARS-CoV-2 spike (S) protein gene among COVID-19 patients’ samples tested with the PCR assay. Samples in which the SARS-CoV-2 nucleocapsid (N) gene was not detected (62 samples) or was detected (25 samples) are compared. In each violin plot, the solid dot indicates the mean *C_T_* value (17.88 and 13.64, respectively) and the solid line indicates the standard deviation value (4.01 and 4.98, respectively). Asterisks indicate a statistically significant difference (*P* < 0.0001) between mean *C_T_* values, as established using a nonparametric Mann-Whitney *U* test.

As shown in Fig. 1, comparison of S-gene *C_T_* values between sample groups revealed that the mean ± SD *C_T_* value of samples with the N gene detected (25 samples; *C_T_*, 13.64 ± 4.98) was significantly lower than that of samples with the N gene not detected (62 samples; *C_T_*, 17.88 ± 4.01) (*P* < 0.0001 by Mann-Whitney *U* test). This led us to hypothesize that a greater RNA template’s amount in those samples could have caused a low level of N-gene PCR amplification even with primer-to-template annealing conditions supposed to be suboptimal.

The role of SARS-CoV-2 N-gene mutations in affecting the diagnostic performance of commercially available SARS-CoV-2 PCR assays remains scarcely explored. One study by Alkhatib et al. (16) reported, for the first time to the authors’ knowledge, N gene target failure using the Seegene Allplex SARS-CoV-2 assay. The case concerned a patient infected with a SARS-CoV-2 Delta sublineage AY.4 variant carrying a G214-G215 deletion in the N gene (one of the PCR assay targets), whose nasopharyngeal swab samples were repeatedly negative for the N gene (16). Interestingly, the patient’s samples gave positive results for E and RNA-dependent RNA polymerase (RdRP)/S genes, mirroring the scenario with SARS-CoV-2 Omicron variant-infected patients presented here. Concomitantly, Chen et al. (17) assessed the impact of six important SARS-CoV-2 variants (Alpha, Beta, Gamma, Delta, Omicron, and Fin-796H) on the analytical sensitivity of the five commercial SARS-CoV-2 PCR assays most used in Chinese laboratories. The authors (17) found that Alpha and Omicron variants had no significant effect on the assays’ detection capability, whereas remarkably, one of these assays failed to detect the N gene in the Fin-796H variant. Not surprisingly, Fin-796H (B.1.1.318), which is not a WHO-designated VOC, was known to carry a mutation in the region targeted by China’s CDC-recommended N-gene assay (17).

In conclusion, while we continue to fear the Omicron variant’s current spread (18), our findings reinforce the importance of ongoing efforts by manufacturers to refine the existing COVID-19 diagnostic PCR assays and/or to develop novel variant-specific PCR assays, thereby making the detection of SARS-CoV-2 variants increasingly rapid and accurate. Despite a fraction of SARS-CoV-2-positive samples, whole-genome sequencing analysis remains crucial to validate PCR assay results. This is particularly true when a PCR assay may no longer be valid for the variant that the assay was designed/used to detect because of newly emergent variants containing mutations outside the PCR assay target region. Finally, as novel variants are bound to emerge, we cannot rule out the risk of SARS-CoV-2 variant misidentification using current PCR assays.
